# Patterns of Cognitive Function Trajectory and Their Associated Factors in Korean Older Women in Single Households

**DOI:** 10.1093/geroni/igaa057.943

**Published:** 2020-12-16

**Authors:** DoKyung Yoon, Hey Jung Jun, Sun Ah Lee

**Affiliations:** Yonsei University, Seoul, Republic of Korea

## Abstract

The aim of this study was to identify distinct patterns of cognitive function trajectory among Korean older women in single households and to investigate their associated factors. We used data from the five waves of the Korean Longitudinal Study of Ageing (KLoSA), a nationally representative longitudinal survey collected biennially from 2006 to 2014. The sample was 244 women aged 65 or above who had maintained the single household status for the five waves. Using Growth Mixture Modeling, distinct patterns of cognitive function trajectory were identified. Then, multinomial regression analysis was employed to examine the associations between identified trajectories and various factors (socio-demographic, health-related, psychological, and interpersonal factors at wave 1). Results showed that there were three distinct patterns of cognitive function trajectory: ‘maintaining-low’ (20.9%), ‘moderate-decreasing’ (33.6%), and ‘maintaining-high’ (45.5%). Specifically, compared to the ‘maintaining-low’ group, younger women were more likely to belong to the ‘moderate-decreasing’ group and the ‘maintaining-high’ group. Furthermore, those with higher household income, and who participated in more various types of social activities (e.g., participation in leisure/culture/sports clubs, volunteering work) were more likely to belong to the ‘maintaining-high’ group. Compared to the ‘moderate-decreasing’ group, those who were younger and participated in more various types of social activities were more likely to belong to the ‘maintaining-high’ group. Findings suggest that socioeconomic status and social engagements may be critical for cognitive function among older women in single households, and encouraging them to remain socially active may help prevent poorer cognitive outcomes.

